# Management of Elderly Patients with Glioblastoma after CE.6

**DOI:** 10.3389/fonc.2017.00196

**Published:** 2017-08-30

**Authors:** Sunit Das, Albert H. Kim, Susan Chang, Mitchel S. Berger

**Affiliations:** ^1^Division of Neurosurgery, Li Ka Shing Knowledge Institute, St. Michael’s Hospital, University of Toronto, Toronto, ON, Canada; ^2^Arthur and Sonia Labatt Brain Tumour Research Centre, Hospital for Sick Kids, Toronto, ON, Canada; ^3^Department of Neurosurgery, Washington University School of Medicine, Siteman Cancer Center, St. Louis, MO, United States; ^4^Department of Neurosurgery, University of California San Francisco, San Francisco, CA, United States

**Keywords:** glioblastoma, elderly patients, temozolomide, chemotherapy, adjuvant, radiation therapy, survival

The introduction of radiation therapy (RT) with concurrent temozolomide (TMZ) chemotherapy resulted in a major shift in the treatment of adult patients with glioblastoma. The EORTC-NCIC trial (the Stupp trial) confirmed significant improvement in overall survival and set the standard of care as 60 Gy in 30 fractions of RT with TMZ daily, followed by 6 months of adjuvant TMZ (1). Further, for the first time, the field of neuro-oncology realized patients with glioblastoma who were achieving longer-term survival, with nearly 40% alive at 2 years, and nearly 10% alive at 5 years (2).

Unfortunately, these successes have not translated into gains for elderly patients with glioblastoma. The EORTC-NCIC data offer little direction regarding best practice for the treatment of elderly patients with glioblastoma, as only a minority of patients enrolled in the Stupp trial were older than age 65, and patients older than 70 were excluded. Further, exploratory analyses of the EORTC-NCIC data suggest that increasing age attenuates the benefit of addition of TMZ to glioblastoma therapy, with less survival benefit among patients 65–70 years of age [hazard ratio for death, 0.78; 95% confidence interval (CI), 0.50–1.24; *P* = 0.29] than among younger patients (3). Meanwhile, the incidence of glioblastoma in the elderly population has been rising (4), bringing with it a growing need to delineate a standard of care for elderly patients.

Anecdotal evidence and previous trial data offered good reason for these patients to have been excluded from the Stupp trial. Many elderly individuals simply cannot tolerate standard RT, let alone combined therapy (5). As it is, the significant biological and functional heterogeneity of this cohort of glioblastoma patients [“not every eighty year old is an eighty year old” (6)] and the many iterations of therapy available their treatment, has resulted in a diverse approach to the care of glioblastoma in elderly patients, as observed in an analysis captured in the SEER registry (7). Defining the standard of care in elderly patients with glioblastoma has been of major interest, but has remained to now an unanswered question.

That treating elderly patients with glioblastoma is appropriate was in itself a question not so long ago. The answer to this question was elucidated by Keime-Guibert and colleagues, who in 2007 published a randomized controlled trial of 85 patients with a Karnofsky performance score of 70 or greater comparing supportive treatment alone to RT (50.4 Gy in 28 fractions) plus supportive care for glioblastoma patients over 70 years of age (the ANOCEF trial) (8). The study was stopped at the first interim analysis due to the finding that survival in the RT plus supportive care group was superior to supportive care alone. Median overall survival for patients who received support care plus radiotherapy was 6.7 months, compared to 3.9 months in patients treated with supportive care alone. Importantly, the study found that the survival benefit offered by RT to elderly patients did not come at the cost of health-related quality of life.

The options for therapy in this population expanded with the 2012 study from the Nordic Brain Tumor Clinical Study Group (the Nordic trial) (9). Nordic randomized 342 patients over 65 years of age with a good performance status (ECOG 0-2) to three single-modality treatment arms: (1) standard-dose TMZ; (2) standard RT (60 Gy in 30 fractions); or (3) hypofractionated RT (34 Gy in 10 fractions). 291 patients underwent treatment with a primary endpoint of overall survival and secondary endpoints of health-related quality of life and safety. Patients deemed eligible for chemoradiation were excluded. The median overall survival was significantly longer in patients treated with TMZ (8.3 months) or hypofractionated RT (7.5 months) compared to those who received standard RT (6.0 months). O^6^-methylguanine–DNA methyltransferase (MGMT) promoter methylation was associated with significantly higher survival rates in patients treated with TMZ (9.7 months vs. 6.8 months), but had no effect on survival in patients treated with RT. No difference in survival was found in patients with an unmethylated MGMT promoter treated with RT or single-agent TMZ (7.0 vs. 6.8 months). Patients in the TMZ group generally reported better quality of life than did patients in the RT groups, but the ratings for global health status were equal.

Perry and colleagues from the CCTG/EORTC Trial Investigators Intergroup have now brought us one step closer to an answer. In CCTG CE.6/EORTC 26062-22061/TROG patients 65 years of age or older with newly diagnosed glioblastoma were randomly assigned to receive either RT alone (40 Gy in 15 fractions) or RT with concomitant and adjuvant TMZ (10). 562 patients were randomized. The median age was 73 years (range, 65–90). Patients deemed by their physicians to be suitable to receive conventional RT were excluded. Eligible patients had an ECOG performance status of 0, 1, or 2 and were receiving glucocorticoids at a stable or decreasing dose. Quality-of-life assessment was performed weekly during RT, then 1 week after the last day of RT, and then every 3 months until disease progression, using the EORTC Quality-of-Life Questionnaire-Core 30 (QLQ-C30) and the EORTC brain module (QLQ-BN20). Progressive disease was defined as objective radiographic progression. If brain imaging could not be performed, symptomatic progression was used to define progression. The primary end point was overall survival, measured from the date of randomization until death or censoring at the last day that the patient was known to be alive. Progression-free survival was measured from the date of randomization until disease progression or death (if no progression was reported) or until the last evaluation date.

All 562 randomly assigned patients (281 in each group) were included in the intention-to-treat analysis. Among the 503 samples examined centrally, glioblastoma was confirmed in 480 (95.4%), high-grade glioma in 15 (3.0%), diffuse glioma lacking high-grade features in 5 (1.0%), and anaplastic oligodendroglioma in 3 (0.6%). Immunohistochemical staining for the IDH1 R132H mutation was positive in only 4 of the 481 specimens deemed suitable for analysis. Treatment adherence was high. The median duration of concomitant TMZ was 21 days, as planned. The median number of adjuvant cycles delivered was five. A similar percentage of patients in the two groups (197 of 493 patients, 40.0%) received other anticancer therapies at disease progression. RT plus TMZ was associated with more adverse events than RT alone, with a higher rate of grade 3 or 4 events, but no difference between the two groups in terms of serious adverse events leading to death.

Baseline factors that correlated with overall survival included the extent of resection and MMSE score: patients with biopsy only had shorter survival than those with partial or complete resection. In Cox regression modeling with adjustment for baseline factors, RT plus TMZ remained significantly better than RT alone with respect to overall survival, with an estimated hazard ratio of 0.67 (95% CI, 0.56–0.80; *P* < 0.001). The median overall survival was longer with RT plus TMZ than with RT alone (9.3 vs. 7.6 months; hazard ratio for death, 0.67), as was the median progression-free survival (5.3 vs. 3.9 months; hazard ratio for disease progression or death, 0.50). Among 165 patients with methylated MGMT status, the median overall survival was 13.5 months with RT plus TMZ and 7.7 months with RT alone (hazard ratio for death, 0.53). Interestingly, even patients with unmethylated MGMT status benefited from the addition of chemotherapy: among 189 patients with unmethylated MGMT status, the median overall survival was 10.0 months with RT plus TMZ and 7.9 months with RT alone (hazard ratio for death, 0.75). Measures of quality of life showed no significant difference in the two trial groups. Further, exploratory analyses of overall survival at 12, 18, and 24 months suggested that the benefit of radiation and concurrent and adjuvant chemotherapy on OS is long standing. Unfortunately, combined therapy in elderly patients does not appear to garner long-term survivorship as it does in younger patients: the CE.6 trial cohort had no survivors beyond 3 years.

The CE.6 trial data, while methodologically sound, were at times scientifically difficult to make sense of. Patients with unmethylated MGMT derived a clinically meaningful if not statistically significant (*P* = 0.55) overall survival advantage from the addition of TMZ to RT. It is difficult to make sense of this outcome biologically, and difficult to reconcile it with findings from the Stupp trial, in which benefit from combined therapy was more pronouncedly realized in MGMT methylated patients. This discrepancy could be an artifact of the assay used in CE.6 to determine MGMT methylation status (real-time methylation-specific PCR), which risks “misclassifying” patients with lower levels of MGMT methylation as unmethylated (11). Further, patients 65–70 years of age derived less benefit from the addition of TMZ than those 71–75 years of age or 76 years of age or older. The CE.6 trial investigators adroitly suggest that this seeming discrepancy could be an indirect result of excluding younger elderly patients who were deemed to be eligible for standard (Stupp protocol) combined chemoradiation; in other words, the CE.6 trial may have been biased to include more robust older elderly patients, while accruing less medically fit younger elderly patients.

Many questions remain to be asked. For example, should medically eligible older patients receive standard (Stupp protocol) combined chemoradiation? And if so, what criteria should be used to determine which elderly patients are medically eligible for standard therapy? Conversely, are there elderly patients who should be treated with palliative RT alone, or some patients who would be better served by treatment with TMZ monotherapy? Finally, which assay should be used to determine MGMT methylation status? These questions will require future work. For now, Perry and colleagues should be congratulated for clarifying the path forward.

## Author Contributions

SD performed literature review and analysis and was involved in the writing of the manuscript. AK, SC, and MB performed critical analysis and editing of the manuscript.

## Conflict of Interest Statement

The authors declare that the research was conducted in the absence of any commercial or financial relationships that could be construed as a potential conflict of interest.
